# Probabilistic inference of short-term synaptic plasticity in neocortical microcircuits

**DOI:** 10.3389/fncom.2013.00075

**Published:** 2013-06-06

**Authors:** Rui P. Costa, P. Jesper Sjöström, Mark C. W. van Rossum

**Affiliations:** ^1^Neuroinformatics Doctoral Training Centre, Institute for Adaptive and Neural Computation, School of Informatics, University of EdinburghEdinburgh, UK; ^2^Department of Neurology and Neurosurgery, The Research Institute of the McGill University Health Centre, McGill UniversityMontreal, QC, Canada; ^3^Institute for Adaptive and Neural Computation, School of Informatics, University of EdinburghEdinburgh, UK

**Keywords:** short-term synaptic plasticity, probabilistic inference, neocortical circuits, experimental design, parameter estimation

## Abstract

Short-term synaptic plasticity is highly diverse across brain area, cortical layer, cell type, and developmental stage. Since short-term plasticity (STP) strongly shapes neural dynamics, this diversity suggests a specific and essential role in neural information processing. Therefore, a correct characterization of short-term synaptic plasticity is an important step towards understanding and modeling neural systems. Phenomenological models have been developed, but they are usually fitted to experimental data using least-mean-square methods. We demonstrate that for typical synaptic dynamics such fitting may give unreliable results. As a solution, we introduce a Bayesian formulation, which yields the posterior distribution over the model parameters given the data. First, we show that common STP protocols yield broad distributions over some model parameters. Using our result we propose a experimental protocol to more accurately determine synaptic dynamics parameters. Next, we infer the model parameters using experimental data from three different neocortical excitatory connection types. This reveals connection-specific distributions, which we use to classify synaptic dynamics. Our approach to demarcate connection-specific synaptic dynamics is an important improvement on the state of the art and reveals novel features from existing data.

## 1. Introduction

Synaptic plasticity is thought to underlie learning and information processing in the brain. Short-term plasticity (STP) refers to transient changes in synaptic efficacy, in the range of tens of milliseconds to several seconds or even minutes (Zucker and Regehr, 2002). It is highly heterogeneous and is correlated with developmental stage (Reyes and Sakmann, 1999), cortical layer (Reyes and Sakmann, 1999), brain area (Wang et al., 2006; Cheetham and Fox, 2010), and postsynaptic cell-type (Markram et al., 1998; Reyes et al., 1998; Scanziani et al., 1998; Tóth and McBain, 2000; Rozov et al., 2001; Sun and Dobrunz, 2006). For instance, short-term depression predominates in the juvenile brain, whereas more mature circuits have a preponderance for short-term facilitation (Pouzat and Hestrin, 1997; Reyes and Sakmann, 1999). Similarly, synapses from neocortical pyramidal cells (PCs) impinging on other PCs are depressing, whereas those onto specific interneurons can be strongly facilitating (Markram et al., 1998; Reyes et al., 1998).

STP has been proposed to shape information processing in neural networks in multiple ways (Abbott and Regehr, 2004; Fung et al., 2012), to enable cortical gain control (Abbott et al., 1997), pattern discrimination (Carvalho and Buonomano, 2011), input filtering (Markram et al., 1998), adaptation (van Rossum et al., 2008), spike burst detection (Maass and Zador, 1999), synchronization (Tsodyks et al., 2000), and to maintain the balance of excitation and inhibition in local circuits (Galarreta and Hestrin, 1998).

To model short-term depression, Tsodyks and Markram (1997) introduced a phenomenological model based on vesicle depletion, here referred to as the Tsodyks–Markram (TM) model. This model was later extended to include short-term facilitation (Markram et al., 1998; Tsodyks et al., 1998). Although several other STP models have been developed (Abbott et al., 1997; Varela et al., 1997; Dittman et al., 2000; Loebel et al., 2009; Pan and Zucker, 2009), the TM model has become particularly popular, probably because of its combination of appealing simplicity and biophysically relevant parameters (Markram et al., 1998; Richardson et al., 2005; Le Bé and Markram, 2006; Wang et al., 2006; Rinaldi et al., 2008; Ramaswamy et al., 2012; Testa-Silva et al., 2012; Romani et al., 2013).

Typically, STP models are numerically fitted to electrophysiological data by least-mean-square algorithms, which yield the parameter values that minimize the error between data and model. However, such fitting algorithms can get stuck in local optima and may provide little information about the certainty of the parameter values. As shown below, such fits may produce inaccurate results and may lead to unreliable clustering. Bayesian inference is a natural alternative, because it yields a *distribution* of parameter values rather than a single outcome. Bayesian inference has recently been applied to neurophysiological data analysis. McGuinness et al. (2010) used this to estimate large and small action potential-evoked Ca^2+^ events, while Bhumbra and Beato (2013) used a Bayesian framework of quantal analysis to estimate synaptic parameters, which required far fewer trials compared to traditional methods. Here, we introduce a Bayesian approach to obtain the posterior distribution of TM model parameters. This enabled us to take into account the uncertainty inherent to experimental data, which provided a more complete description of STP data.

Our approach has several advantages. First, it allowed us to infer the distribution of synaptic parameters for individual connections and propose a better protocol to extract these parameters. Second, we found that parameter distributions extracted from cortical data are specific to different connection types. Third, we showed that we can automatically cluster the parameters of synaptic dynamics to at least partially classify postsynaptic cell types. We also performed model selection to determine which variant of the TM model best captures the synaptic dynamics of the connection type at hand.

## 2. Materials and methods

### 2.1. Short-term plasticity phenomenological model

The extended TM model (eTM) is a phenomenological model of short-term plasticity defined by the following ODEs (Markram et al., 1998; Tsodyks et al., 1998)

(1) $$\frac{dR(t)}{dt}=\frac{1-R(t)}{D}-u(t^{-})R(t^{-})\delta (t-t_{AP})$$

(2) $$\frac{du(t)}{dt}=\frac{U-u(t)}{F}+f[1-u(t^{-})]\delta (t-t_{AP})$$

The first equation models the vesicle depletion process, where the number of vesicles *R*(*t*) is decreased with *u*(*t*)*R*(*t*) after release due to a presynaptic spike at time *t*_*AP*_, modeled by a Dirac delta distribution δ(*t*). Between spikes *R*(*t*) recovers to 1 with a depression timeconstant *D*. The second equation models the dynamics of the release probability *u*(*t*) which increases with *f*[1 − *u*(*t*)] after every presynaptic spike, decaying back to baseline release probability *U* with a facilitation timeconstant *F*. The notation *t*^−^ indicates that these functions should be evaluated in the limit approaching the time of the action potential from below (as would be natural in forward Euler integration).

By varying the four parameters $\vec{\theta }=\{D,F,U,f\}$ one can obtain depressing, combined facilitating-depressing and facilitating synaptic dynamics. We note that for some data a three parameter model [setting *f* = *U*, denoted the TM with facilitation model] or even a two parameter depression model with only Equation (1) [setting *u*(*t*) = *U*, denoted the TM model] is sufficient. This, however, is not generally the case, as shown below.

To speed up the numerical implementation we integrated the above equations between spikes *n* and *n* + 1, a time Δ*t*_*n*_ apart, yielding

(3) $$R_{n+1}=1-\left[1-R_{n}(1-u_{n})\right]\exp\left(-\frac{\Delta t_{n}}{D}\right)$$

(4) $$u_{n+1}=U+\left[u_{n}+f(1-u_{n})-U\right]\exp\left(-\frac{\Delta t_{n}}{F}\right)$$

As we assumed that at time zero the synapse has not been recently activated, we set *R*_0_ = 1 and *u*_0_ = *U*.

The postsynaptic potential PSP_*n*_ is given by

(5) $${\text{PSP}}_{n}=AR_{n}u_{n}$$

where *A* is an amplitude factor that includes the number of release sites, the properties and number of postsynaptic receptors, and cable filtering.

The steady-state values *R*_∞_ and *u*_∞_ in response to prolonged periodic stimulation with rate ρ are

(6) $$R_{\infty }(\rho )=\frac{1-\exp\left(-\frac{1}{\rho D}\right)}{1-\left[1-u_{\infty }(\rho )\right]\exp\left(-\frac{1}{\rho D}\right)}$$

(7) $$u_{\infty }(\rho )=\frac{U+(f-U)\exp\left(-\frac{1}{\rho F}\right)}{1-(1-f)\exp\left(-\frac{1}{\rho F}\right)}$$

### 2.2. Simulated data

For the simulated data we used five sets of STP parameters, ranging from depression to facilitation, see Table 1.

**Table 1:** **The five parameter sets used for simulated data**.

**Synaptic dynamics regime**	**D(s)**	**F(s)**	**U**	**f**	**EPR**
Strong depression	1.70	0.02	0.7	0.05	0.45
Depression	0.50	0.05	0.5	0.05	0.64
Facilitation-depression	0.20	0.20	0.25	0.3	0.94
Facilitation	0.05	0.50	0.15	0.15	1.26
Strong facilitation	0.02	1.70	0.1	0.11	1.43

EPR was calculated by simulating the eTM model with 5 pulses at 30 Hz as shown in Figure 2.

As the commonly used paired-pulse ratio, PPR = PSP_2_/PSP_1_, only takes the first two pulses into account, we introduce the Every Pulse Ratio (EPR) as a more comprehensive measure of STP dynamics. It is defined as

(8) $$\text{EPR}=\frac{1}{\left(n-1\right)}\sum_{i=1}^{n-1}\frac{{\text{PSP}}_{i+1}}{{\text{PSP}}_{i}}$$

This index measures the average amplitude change from the *i* to the *i* + 1 response normalized to the *i* response in the train. EPR is used in Table 1 and elsewhere to quantify the average degree of depression (EPR < 1) or facilitation (EPR > 1). Using these parameters we calculated the synaptic responses with Equations (3, 4) to a spike train of five pulses at 30 Hz (Figures 2, 4).

### 2.3. Bayesian formulation

**Figure 1:**
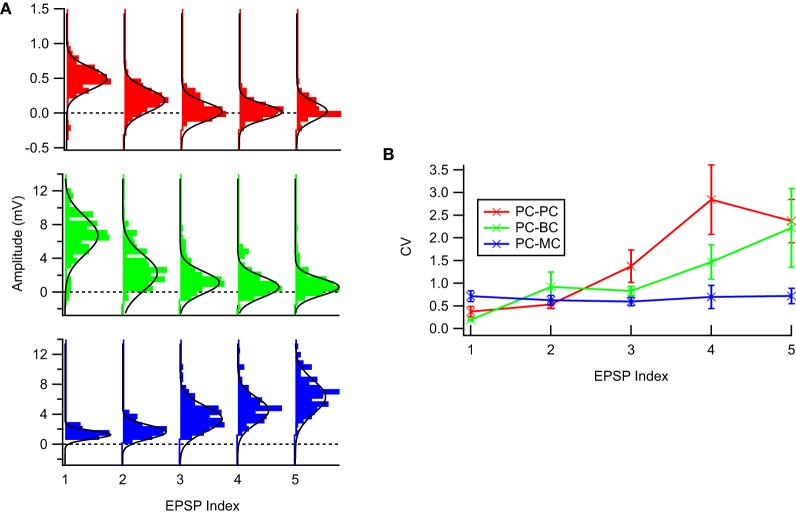
**The experimental STP data was well described by a Gaussian noise model. (A)** Sample EPSP distributions for the three connection types: PC–PC (top, red), PC–BC (middle, green), and PC–MC (bottom, blue) with respective Gaussian fits (solid black line)—94% of the EPSP distributions were not statistically significant different from a Gaussian distribution (see main text for more details). **(B)** Coefficient of variation analysis. While for facilitating synapses (PC–MC) it was more or less constant, for depressing synapses (PC–PC and PC–BC) we observed an approximately linear increase with EPSP amplitude. Error bars represent standard error of the mean.

The posterior distribution of the synaptic parameters follows from Bayes' theorem as $P(\vec{\theta }|\vec{d})\propto P(\vec{\theta })P(\vec{d}|\vec{\theta })$, where $\vec{d}$ is a vector of mean postsynaptic potential peaks extracted from simulated or experimental data and $\vec{\theta }$ is a vector encompassing the model parameters. Many factors contribute to variability in the measured EPSPs, including stochastic vesicle release and experimental noise. A typical noise model of synaptic transmission is a binomial distribution (Zucker and Regehr, 2002). However, we found that our data is well described by a Gaussian noise model (see below). Therefore, we write the likelihood of the data as

(9) $$P(\vec{d}|\vec{\theta })=\prod_{i=1}^{N}\frac{1}{\sqrt{2\pi \text{​}\sigma_{i}^{2}}}\exp\left[\text{​}-\text{​}{\left(d_{i}-\text{STP}\left({\text{PSP}}_{i}|\vec{\theta }\right)\right)}^{2}\text{​​}/2\sigma_{i}^{2}\right]$$

where $\text{STP}({\text{PSP}}_{i}|\vec{\theta })$ is the voltage response from the eTM model for *i* = 1…*N* runs over the data points in the pulse train. We set the noise σ_*i*_ independently for each pulse. For the data we extracted the CV for each pulse, while for the simulated data a fixed coefficient of variation (CV = 0.5) was assumed, based on Figure 1. Note that we did not include a model of stochastic vesicle release. This would be a possible extension of our model. A stochastic release model also leads to correlations between subsequent events, and Equations (4, 3) would thus have to be extended to their history-dependent variances, which would complicate our model. We did confirm that parameters from a simulated stochastic release model, were inferred correctly using the above noise model, although the posterior distributions were somewhat widened.

The priors were modeled as independent non-informative flat distributions over the model parameters

(10) $$P(\vec{\theta })=\{\begin{array}{l} P(D)=P(F)=\text{Uniform}[0,2]\\ P(U)=P(f)=\text{Uniform}[0,1] \end{array}$$

which limits the posterior distribution within reasonable values.

Bhumbra and Beato (2013) sampled their bidimensional posterior probability using a brute-force grid search. For higher dimensions this is computationally expensive. We therefore inferred the posterior distribution by sampling using the Slice Sampling Markov Chain Monte Carlo (MCMC) method (Neal, 2003). The width parameter *w* was set equal to the upper limit of the flat prior distributions (i.e., $\vec{w}=\{2,2,1,1\}$) and each parameter is sampled sequentially in the four orthogonal directions. We discarded the first 2500 samples as burn-in and use the last 7500. For the numerical implementation we use the log-likelihood $\log P(\vec{d}|\vec{\theta })$. The convergence of the Markov chain to the equilibrium distribution was assessed through the Gelman–Rubin statistical method (Brooks and Gelman, 1998). However, this diagnostic of convergence can indicate lack of convergence, but does not confirm it. Therefore, in order to ensure convergence, we used multiple chains (*n* = 3) starting at different initial conditions to ensure that the outcome was independent on the initial condition (Gelman and Shirley, 2011). The maximum *a posteriori* (MAP) estimator of the synaptic parameters is given by

(11) $${\vec{\theta }}_{\text{MAP}}={\text{argmax}}_{\vec{\theta }}P(\vec{\theta })P(\vec{d}|\vec{\theta })$$

The MAP estimate was obtained by keeping the most likely sample from multiple MCMC chains. In addition we also ran an optimizer to find the most precise MAP using the distribution peak as a starting point. As both approaches gave equally good fits for the sake of simplicity we decided to use the former.

We compared our estimation method with a standard stochastic optimization method, simulation annealing (SA). The SA method minimizes the RMSE

(12) $${\vec{\theta }}_{SA}={\text{argmin}}_{\vec{\theta }}\sqrt{\frac{1}{N}\sum_{i=1}^{N}{\left[d_{i}-\text{STP}\left({\text{PSP}}_{i}|\vec{\theta }\right)\right]}^{2}}$$

while trying to avoid getting stuck in local minima. We ran the SA algorithm 200 times and selected the estimate with lowest RMSE. Using an objective function scaled by the variance gave similar results when compared to the non-scaled version; thus for the sake of comparison with previous literature, we used the non-scaled version. To compare the goodness of fit of both MAP and SA solutions with the data, we used the coefficient of determination *R*^2^.

As the amplitude *A* is not relevant for the synaptic dynamics, we set *A* = *A*^MAP^,

(13) $$A^{\text{MAP}}=\frac{\sum_{i=1}^{N}d_{i}m_{i}/\sigma_{i}^{2}}{\sum_{i=1}^{N}m_{i}^{2}/\sigma_{i}^{2}}$$

where $m_{i}=\text{STP}({\text{PSP}}_{i}|\vec{\theta })$. We used this value to normalize the data. Its value does not affect the dynamics estimation, because *A* only scales the responses.

To estimate the posterior probability distributions, we used a kernel density estimation method (Ihler and Mandel, 2007). Unless otherwise stated, the code was implemented in Matlab (inference code is available online^1^).

#### 2.3.1. Quantifying inference performance

To quantify which protocol allows for the most precise recovery of the true parameters of simulated STP data (Figure 3A), we computed the sample estimation error over *N* = 22,500 MCMC samples $\vec{\theta }$ to the true parameters ${\vec{\theta }}^{*}$, as $E=\langle {\sum_{i=1}^{4}\left[(\theta_{i}-\theta_{i}^{*})/\theta_{i}^{*}\right]}^{2}\rangle$, where the average is over all the runs and all five parameter sets (Table 1). To achieve similar weighting, the parameters were normalized to the true parameters. Alternatively, we normalized the estimated parameters on the upper limit of their priors, or we omitted normalization altogether. This yielded similar results. Note that in probabilistic spirit, this error also quantifies the spread in the distribution. A smaller *E* gives more peaked distributions, which correspond to tighter parameter estimates. Note that, although similar, this error measure does not follow the standard bootstrap approach.

#### 2.3.2. Model selection

For model selection, we used the Akaike Information Criterion (AIC), which is a information-theoretic measure of the goodness of fit of a given statistical model. It is defined as $AIC=2k-\log P({\vec{\theta }}_{\text{MAP}}|\vec{d})$, where *k* is the number of estimable parameters in the model and $\log P({\vec{\theta }}_{\text{MAP}}|\vec{d})$ the log-posterior of the MAP estimate on the normalized data. The AIC evaluates models according to their parsimonious description of the data, and is particularly suitable for probabilistic inference. We used the evidence ratio, which is a relative ranking of the Akaike weights, to find the least complex model that best describes the data (Turkheimer et al., 2003; Nakagawa and Hauber, 2011).

### 2.4. Electrophysiology

Quadruple whole-cell recordings and extracellular stimulation were performed in acute visual cortex slices of young mice (P12–P20) as previously described (Buchanan et al., 2012). The stimulating electrode was positioned in layer 5 (L5). L5 Pyramidal cells (PCs) were targeted based on their characteristic pyramidal soma and thick apical dendrite. Basket cells (BCs) were targeted in transgenic mice genetically tagged for parvalbumin, while Martinotti cells (MCs) were targeted in mice genetically labeled for somatostatin (Markram et al., 2004; Buchanan et al., 2012). Cell identities were verified by cell morphology and rheobase firing pattern. Five spikes were elicited at 30 Hz using 5 ms long current injections (0.7–1.4 nA) every 18 s in all neurons throughout the experiment. Excitatory postsynaptic potentials (EPSPs) were averaged from 20–40 sweeps.

For each connection, a histogram was built from the EPSP amplitudes extracted with 1–2-ms window fixed approximately on the peak depolarization. EPSP distributions were fit with a Gaussian (Equation 9). Recordings with mean EPSPs smaller than 0.015 mV were discarded. Electrophysiological data analysis was carried out in Igor Pro (WaveMetrics Inc., Lake Oswego, OR).

Figure 1 shows typical EPSP distributions for each of the three neocortical excitatory connection types that we studied, PC–PC, PC–BC, and PC–MC. We tested whether the Gaussian noise model was a valid description of the data using the Kolmogorov–Smirnov (KS) normality test, and we found that the null hypothesis that samples were drawn from a normal distribution could not be rejected for 160 out of 170 EPSP distributions, with no connection-specific bias. This suggests that EPSPs were typically normally distributed, consistent with previously published results [e.g., Figure 5B in Markram et al. (1997)]. Due to noise, apparently negative EPSPs (Figure 1) were occasionally recorded. These are consistent with our Gaussian noise model and require no special treatment.

### 2.5. Clustering and classification

Distributional clustering was introduced by Pereira et al. (1993). Here we applied a similar information-theoretic approach to cluster $P(\vec{\theta }|\vec{d})$. Instead of a “soft” clustering approach we used “hard” clustering, due to its simplicity, computation speed and comparison with standard clustering techniques. We used an agglomerative method [unweighted average distance method, Sokal (1958)] and an f-divergence metric. F-divergence metrics constitute a family of functions that measure the difference between two probability distributions. Consider two discrete probability distributions *P* and *Q* both discretized into *N* bins. To compare any given pair of distributions we used two f-divergence metrics: (i) the symmetrized Kullback–Leibler divergence

(14) $${\text{KL}}_{s}(P,Q)=\frac{\text{KL}(P,Q)+\text{KL}(Q,P)}{2}$$

with

(15) $$\text{KL}(P,Q)=\sum_{i=1}^{N}P_{i}(\vec{\theta }|\vec{d})\log\frac{P_{i}(\vec{\theta }|\vec{d})}{Q_{i}(\vec{\theta }|\vec{d})}$$

and the (ii) Hellinger distance

(16) $$\text{HL}(P,Q)=\frac{1}{\sqrt{2}}\sqrt{\sum_{i=1}^{N}{\left(\sqrt{P_{i}(\vec{\theta }|\vec{d})}-\sqrt{Q_{i}(\vec{\theta }|\vec{d})}\right)}^{2}}$$

Due to the high dimensionality of our problem, we approximated these two measures first marginalizing $P(\vec{\theta }|\vec{d})$ over the *d* = 4 dimensions and then computing the sKL and HL over the *d* marginal probabilities. We compared our posterior-based clustering with clustering based on the SA estimates. Here, we used the Euclidian distance on the z-scored parameters found with SA.

To estimate the number of clusters we used the Pseudo-F statistic (Caliński and Harabasz, 1974). The Pseudo-F statistic captures the tightness of clusters as the ratio of the mean sum of squares between clusters to the mean sum of squares within cluster

(17) $$\text{Pseudo-F}=\frac{\left(T-P_{G})/(G-1\right)}{P_{G}/(n-G)}$$

where $T=\sum_{i=1}^{n}{\left(P_{i}-\bar{P}\right)}^{2}$ is the total sum of squares, $P_{G}=\sum_{i=1}^{G}\sum_{j=1}^{n_{i}}{\left(P_{i}^{j}-\bar{P_{i}}\right)}^{2}$ is the within-cluster sum of squares, *G* is the number of clusters, and *n* the total number of items. A larger Pseudo-F usually indicates a better clustering solution. The Pseudo-F statistic has been found to give best performance in simulation studies when compared with 30 other methods (Milligan and Cooper, 1985).

To evaluate the clustering quality, we computed the dendrogram purity as described by Heller and Ghahramani (2005), where we considered two classes according to EPR: class 1 for EPR ≤ 1 and class 2 for EPR > 1. This threshold allows us to separate mostly depressing from mostly facilitating synaptic dynamics.

Finally, we also performed classification using the Naive Bayes Classifier: $P(C|\vec{\theta })\propto P(C)P(\vec{\theta }|C)$, where *P*(*C*) is the prior over the different synapse types *C* and $P(\vec{\theta }|C)$ the likelihood for a given class. Although information about connectivity rates could in principle be incorporated in the prior, we used a uniform prior over the classes. Our likelihood is given by the MCMC inference over the model parameters for a given training dataset *d*_*C*_ and synapse type *C*, i.e., $P(\vec{\theta }|C)=P(\vec{\theta }|d_{C})$. As the Naive Bayes Classifier assumes independence between the different classes, we have one independent model per class with the maximum a posterior decision rule ${\text{argmax}}_{\left(c\in C\right)}P(C=c)P({\vec{\theta }}_{\text{MAP}}|C=c)$. We estimated the performance of our classifier with K-cross validation (*K* = 7, i.e., ~80% for PC–PC (*n* = 9) and PC–MC (*n* = 9), and ~60% for PC–BC (*n* = 12)), where we sampled over K data points (i.e., connections) for each synapse-type to obtain our likelihood model and then test the classifier with the remaining data points. This process was repeated until all possible different K partitions of the data have been used. Accuracy is defined as the percentage of correct classifications for a given connection type.

## 3. Results

### 3.1. Parameter inference certainty is synaptic dynamics dependent

**Figure 2:**
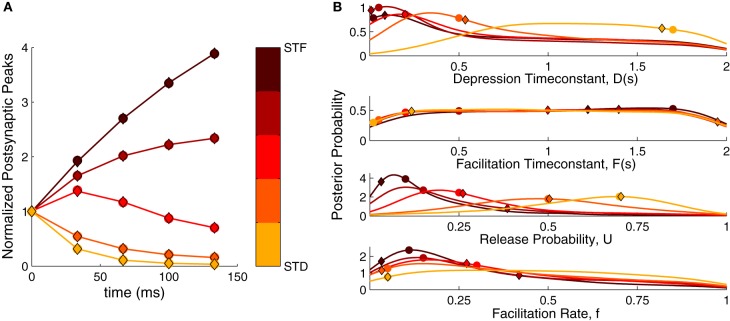
**Bayesian inference of short-term plasticity parameters using simulated data. (A)** Simulated PSPs (filled circles in response to five pulses at 30 Hz) for five different synaptic parameter settings ranging from strong depression (yellow) to strong facilitation (dark red). The MAP solution of the inferred distribution is shown with diamonds. **(B)** Posterior marginalized distributions of the model parameters for the data in **(A)**. The true parameters are shown as filled circles and the MAP solutions as diamonds.

We first checked our method in extracting STP parameters from simulated data with a standard stimulus train of five spikes at 30 Hz (see Materials and Methods). We simulated data with predefined parameter sets ranging from strong depression to strong facilitation. This was achieved by decreasing the baseline release probability *U* and the depression timeconstant *D*, while increasing the facilitation rate *f* and the facilitation timeconstant F (see Materials and Methods, Table 1). The resulting dynamics are shown in Figure 2A.

Figure 2B shows the inferred parameter distributions for the various parameter settings. As the full posterior distribution is four dimensional, we plotted the marginals only. The inferred parameter distributions showed varying behavior: The distributions for *U* were well-tuned to values close to the true parameter values. For the *D* parameter the shifts in the distributions followed the changes in the true parameter, becoming broader for depressing dynamics. Both *F* and *f* were not narrowly tuned to the true parameter. Although *f* was tuned to small values for facilitating synapses, its distribution became broader for depressing synapses. The *F* parameter was not particularly tuned to any value, being close to an uniform distribution for both depressing and facilitating synapses. We explored the possibility that the broadness of F depended on the prior boundary by extending it to 5 s and 10 s. However, the distribution remained uniform and merely grew wider, suggesting that the broad distribution was not caused by an improper choice of prior. In summary, the inference procedure shows that—depending on the dynamics—the inferred parameter distributions can be narrow or broad and that some parameters are much more tightly constrained than others.

### 3.2. Improving experimental protocol for parameter inference

**Figure 3:**
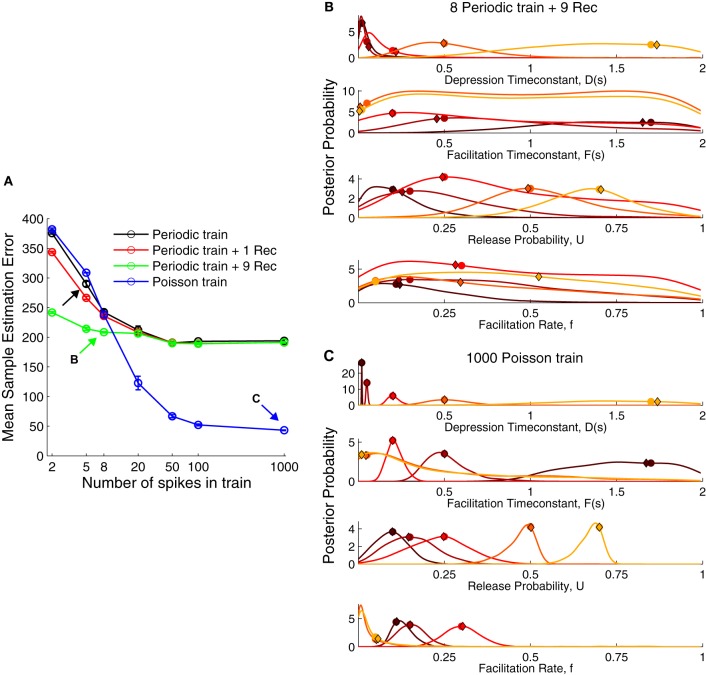
**The performance of various stimulation protocols to infer short-term plasticity parameters. (A)** Comparison of mean sample estimation error using different stimulation trains. Black arrow corresponds to the protocol used in Figure 2. A periodic spike train at 30 Hz with eight pulses and nine recovery pulses [green arrow, (B)] already provided a better estimate of the STP parameters. However, a Poisson train provided a much smaller error when using more than 20 spikes with a close to zero error for 1000 spikes [blue arrow, (C)]. **(B)** Posterior distribution for a periodic train with nine recovery pulses (cf. Figure 2B). **(C)** Posterior distribution for a Poisson train with 1000 pulses. The true parameters are shown as filled circles and the MAP solutions as diamonds. For visualization the marginal probabilities were scaled by their standard deviation.

The fact that some of the inferred parameter distributions were broad suggested that the five pulse protocol did not yield enough information to reliably infer the true parameters. Therefore, we used our probabilistic formulation to find an experimental protocol that improves the inference quality (Figure 3). To this end, we compared the sample estimation error on the estimates (see Materials and Methods) for different spike trains: (1) a periodic train at 30 Hz, (2) a periodic train with recovery pulses, and (3) a Poisson train of 30 Hz (Figure 3A). We also varied the number of spikes in the train.

The widely used paired-pulse protocol to probe synaptic dynamics gave poor estimates even when coupled with nine recovery pulses spaced exponentially across 4 s. Using five pulses in the spike train improved the performance only moderately. Some studies have inferred the TM model parameters with eight spikes and a single recovery pulse after 500 ms (e.g., Wang et al., 2006). This did not improve the recovery error when compared to a periodic spike train alone. A Poisson spike train, however, surpassed other protocols using only 20 spikes. Therefore, we propose a Poisson spike train with 20…100 spikes as a better protocol to obtain accurate estimates of the model parameters. However, also a spike train with eight periodic pulses and nine recovery pulses offers a good compromise, yielding a low recovery error in a reasonably short duration (≈4.23 s). The distributions for these two protocols were more narrowly tuned to the true parameters (Figures 3B,C) compared to a periodic spike train without a full recovery phase (Figure 3B). Contrary to our intuition, the distributions for *D* were more narrowly tuned for facilitation (darker colors) than for depression (lighter colors). Although for the sake of simplicity, we do not show the results for a short periodic train followed by a Poisson train, such an approach would combine the ability to compute standard STP measures and recover information across frequencies. The reason for the poor performance of periodic trains even with many pulses is that the synapse quickly reaches steady-state, given by Equations (6, 7). Hence additional pulses do not increase information and the estimation error quickly reaches a plateau. In contrast, a random Poisson train allows the inference process to converge to the true parameter distributions in the limit of large spike trains.

Note, that both in Figure 2B and Figures 3B,C, the MAP solution is not always at the peak of the marginal distributions. The reason is that when there are dependencies in the parameters, the peak in the full distribution $P(\vec{\theta })$ does not need to coincide with the peaks of the marginals. Indeed, when we compared the log-posterior of the MAP estimate to the log-posterior of the estimate given by the maximum of each marginal probability alone, the MAP approach yielded a much better estimate: $\log P({\vec{\theta }}_{\text{MAP}}|\vec{d})=-0.0038$, compared to the maximum of the marginal probabilities, $\log P({\vec{\theta }}_{\text{marginals}}|\vec{d})=-0.6588$ .

### 3.3. Probabilistic inference of neocortical data reveals connection-specific distributions

**Figure 4:**
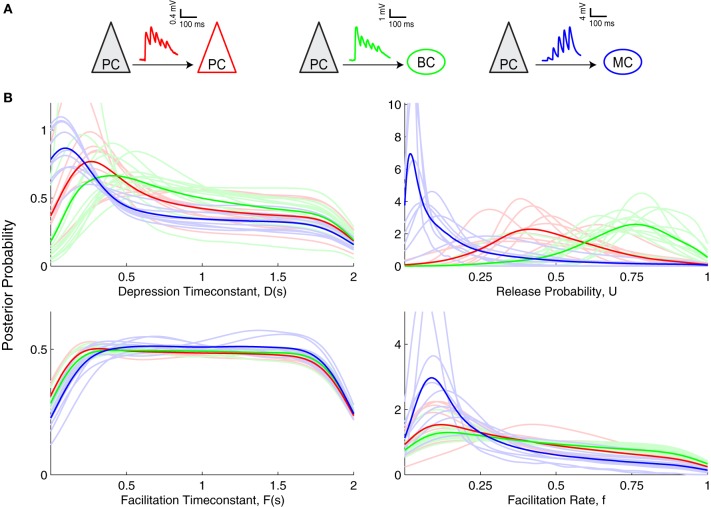
**Inference of short-term plasticity parameters from experimental data from visual cortex. (A)** Sample experimental STP traces are shown for PC–PC (red), PC–BC (green), and for PC–MC (blue) connections. **(B)** Marginalized posterior distributions obtained using slice sampling from these three different excitatory connections suggest that PC–MC (*n* = 9) connections are quite different from PC–PC (*n* = 9) and PC–BC (*n* = 12) connections. Light colored lines show individual connections, while dark colored lines correspond to their average.

Next, we performed Bayesian inference of the eTM parameters on experimental data from visual cortex L5. These data was recorded earlier using a standard five-pulse protocol, instead of the improved protocols suggested above. This means that the parameters may not be optimally constrained, but the overall findings should still hold. We inferred the posterior distributions of the parameters *U, D, F*, and *f* from PC–PC, PC–MC, and PC–BC connections (Figure 4A).

When comparing the Bayesian model inference of these three different synapse types (Figure 4B), the most salient difference was observed in the *U* parameter, i.e., the baseline probability of release. PC–MC connections had a small *U, D* and *f*. PC–PC connections had a medium *U*, medium to high *D*, a close to uniform *F* and a broad *f* with a preference for smaller values. PC–BC connections were similar to PC–PC connections, apart from a larger *U* (PC–BC: 0.72 ± 0.04, *n* = 12; PC–PC: 0.53 ± 0.05, *n* = 9; *p* < 0.01, Mann–Whitney test based on the MAP estimates). This higher value of *U* indicates that PC–BC synapses are generally more strongly depressing than PC–PC synapses. However, the EPRs for these two connection types were indistinguishable (PC–BC: 0.63 ± 0.04, *n* = 12; PC–PC: 0.69 ± 0.03, *n* = 9; *p* = 0.21, Mann–Whitney test), suggesting that the model inference is more sensitive than the EPR measure, and is therefore better suited for picking up connection-specific differences in STP.

**Figure 5:**
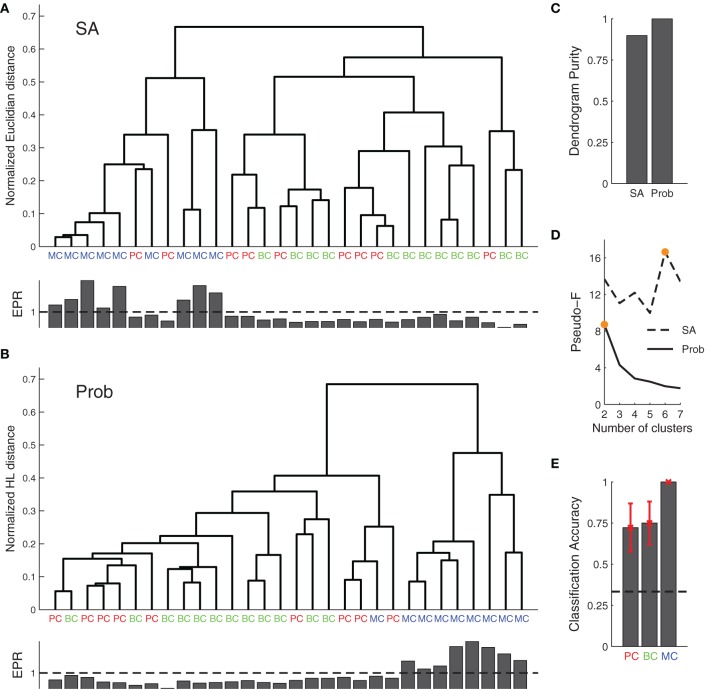
**Agglomerative clustering using posterior distributions improves synaptic dynamics clustering. (A)** Clustering based on the synaptic parameters found by SA did not produce good clusters. **(B)** Clustering of posterior distributions using the probabilistic approach with the Hellinger distance gave rise to two clusters: one for short-term depression and the other for short-term facilitation (cf. EPR, inset bottom), with the first corresponding to both PC–PC and PC–BC connections, while the other roughly mapped onto PC–MC synapses. **(C)** EPR-based dendrogram purity with probability distribution-based clustering is higher than the purity from SA-based clustering. **(D)** Maximal Pseudo-F statistic suggests that the data contains two or six clusters when clustering the posterior distributions or SA-based clustering, respectively (orange filled circles). **(E)** A simple probabilistic classifier (Naive Bayes) achieved good performance for all the connection types, in particular for PC–MC connections (black dashed line represents chance level). Error bars represent standard error of the mean.

We next used our Bayesian approach for synapse classification. We first clustered the data of the various connections based on the model parameters found by SA, Figure 5A. We next clustered based on the marginalized posterior distributions, Figure 5B using the Hellinger distance (see Materials and Methods). Clustering analysis showed that the Bayesian approach improved the dendrogram purity (Figure 5C), as it split the data into two distinct clusters as assessed by the Pseudo-F statistic (Figures 5B,D).

With SA-based clustering, the Pseudo-F statistic suggested six clusters (Figure 5D) with a lower dendrogram purity (Figure 5C, 0.89 purity level), which indicates that these six clusters are spurious. Furthermore, with the Bayesian approach, the clusters map better to the EPR measure (Figure 5B, inset bottom), indicating that our approach captures the synaptic properties better than the SA approach. The two clusters found by our approach correspond to synapses that are either chiefly depressing or facilitating. Still, the clusters did not correspond well to synapse type. In particular, PC–PC and PC–BC synapses were classified as the same type.

In an alternative approach, we also clustered the Bayesian posteriors using the symmetric KL-divergence (sKL). The sKL achieved 0.78 dendrogram purity and three clusters according to the Pseudo-F statistic; thus performing worse.

To determine how well the posterior distributions could be classified in keeping with the three connection types, we performed Naive Bayes classification with a 7-fold cross-validation (Figure 5E). We obtained 100% accuracy in PC–MC connection classification. Surprisingly, however, we also obtained a 72% and 75% classification accuracy for PC–PC and PC–BC connections, respectively. These results suggest that each synapse type can be to some extent separated from the other two types. The ability to separate the different connection types is likely to be mostly due to differences in the baseline release probability (cf. Figure 4B, parameter U).

### 3.4. Comparison to traditional fitting methods

**Figure 6:**
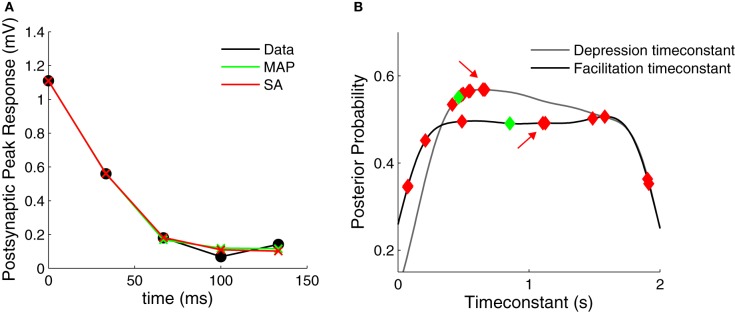
**Comparison of Bayesian approach and traditional fitting methods. (A)** STP models using either MAP or SA solutions (green and red crosses, respectively) provide good fits to the experimental data (black filled circles). **(B)** Marginalized posterior distributions for the depression and facilitation timeconstants (gray and black line, respectively). When fitting the data in **(A)** 10 times, SA yields widely different parameter values (red diamonds, all solutions provided good fits to the data *R*^2^ > 0.99). The MAP solution is shown with green diamonds. The red arrows indicate the SA fit used in **(A)**.

Above we found that for both the simulated and the experimental data, the marginalized posterior of the *F* parameter resembles an uniform distribution (Figures 2B, 4B). This suggests that standard fitting techniques might not perform well and may become trapped in local minima, thus explaining why the SA-based clustering is not able to separate the different synaptic dynamics as well. To test this idea, we used SA on a depressing PC–PC connection and we found that this was indeed the case (Figure 6). Although the method found everytime good fits to the data (Figure 6A), the fit parameters were highly variable from one run to the next (Figure 6B). Although this variability could be used as a proxy for the parameter variance, there is no principled way in SA to estimate parameter variance. In contrast, with our Bayesian approach, the variability and exact distribution is captured in the posterior distribution. Similar observations were made by Varela et al. (1997), who occasionally found an elongated error valley when fitting their particular STP model.

### 3.5. Finding the best model using probabilistic inference

**Figure 7:**
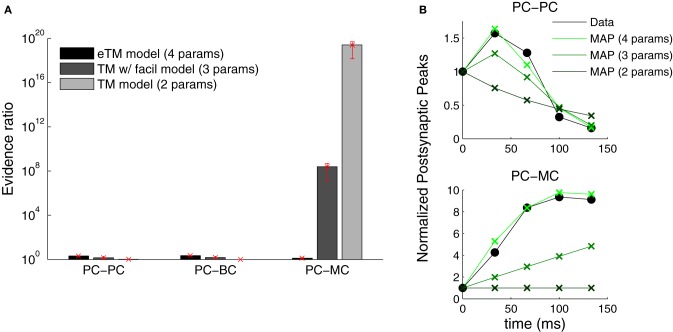
**Extended TM model improves the fitting of PC–MC synapses. (A)** The evidence ratio based on the Akaike Information Criterion is plotted on a logarithmic scale for the three excitatory synaptic types. Three formulations of the Tsodyks–Markram model were compared—with only depression (TM, two parameters), with a degree of facilitation (TM with facilitation, three parameters) and the extended version with full facilitation (eTM, four parameters). Error bars represent standard error of the mean. **(B)** Examples of normalized postsynaptic peak responses that can only be accurately fitted by the eTM model. **Top:** PC–PC recording with combined depression and facilitation. **Bottom:** PC–MC recording with attenuating facilitation. The postsynaptic peak responses (black filled circles) are given together with the MAP solutions from the two, three, and four parameters model, from dark to light green, respectively.

The Bayesian approach offers a natural way to examine which model describes the data most parsimoniously. We performed model selection to identify which formulation of the TM model better described the data (see Materials and Methods). We compared three formulations of the TM model: (1) with depression only—only Equation (1) with *D* and *U* (two parameters)—, (2) depression and facilitation—Equations (1, 2) with *D, F* and *U* (three parameters)—and, (3) the full extended model used above. Figure 7 shows that only the extended model is able to account for all the data from the three connection types. In contrast to Markram et al. (1998) and Richardson et al. (2005), we found that the TM-with-facilitation model does not fit the PC–MC connections well. Although for some recordings the three-parameter model was sufficient, it failed to fit other recordings (Figure 7B). This discrepancy might be due to experimental differences; our dataset was recorded in mice visual cortex L5 and included extracellular stimulation experiments, while Markram et al. (1998) and Richardson et al. (2005) recorded in the somatosensory cortex of the rat using paired recordings only.

## 4. Discussion

Past studies characterizing short-term synaptic dynamics have typically used traditional fitting methods. A Bayesian approach, however, turns out to be particularly advantageous for this problem, because accurate estimation of synaptic parameters is complicated. Here, we have shown that—depending on the synaptic dynamics and experimental protocol—some parameters are not narrowly tuned but broadly distributed. This insensitivity may cause traditional least-mean-square methods to get stuck in local minima.

When applied to experimental data, our method showed that different connections have different distributions. Such synapse-type specific plasticity supports the idea that different synapses perform different computations and subserve different functional roles in the local circuit. Our approach more robustly classifies synapses according to their synaptic dynamics than does clustering using simple point estimates of parameters obtained from standard optimization techniques. Our method might thus enable automatic and independent classification of synapses and cells taking into account the natural variability in the data. Future studies using larger datasets may better identify the synaptic properties that are specific to individual clusters. Furthermore, a model with a more detailed noise description could allow us to also infer the quantal parameters, which could in principle be combined with the Bayesian quantal analysis framework (Bhumbra and Beato, 2013).

We found that inference of the model parameters can be improved by having more pulses as well as by including a recovery phase. The data used here, however, was collected using a standard STP electrophysiology protocol with five pulses at 30 Hz, which still enabled connection-specific clustering. To improve parameter inference further, we propose a combination of a periodic spike train and a Poisson spike train. More pulses add more information, which has an unsurprising positive impact on inference. Poisson trains cover the frequency space better without requiring excessively long experimental recordings. Indeed, Poisson trains add a considerable improvement as compared to the more standard protocol of using fixed-frequency trains (Markram and Tsodyks, 1996; Sjöström et al., 2003).

Experimentally STP has been observed to change with development (Reyes and Sakmann, 1999), drug wash-in (Buchanan et al., 2012), temperature changes (Klyachko and Stevens, 2006), and plasticity (Markram and Tsodyks, 1996; Sjöström et al., 2003). In such situations, it often becomes important to ascertain the particular parameter changes that occur. The Bayesian framework introduced here can be extended to elucidate which components of STP are affected by integrating prior knowledge, through an informative prior. For instance, inferred distributions can be tracked across development.

Our work can also be applied in constructing computer network models with STP using posterior distributions inferred from actual biological data as a generative model. This would yield models with richer dynamics without resorting to simplistic and unrealistic *ad-hoc* approaches to generate synaptic variability that are poorly grounded in biological data.

Our Bayesian approach promises improved computer models as well as a better and more nuanced understanding of biological data. Yet, this approach is not computationally intense, nor is it difficult to implement. We therefore fully expect probabilistic inference of STP parameters to become a widespread practice in the immediate future.

### Conflict of interest statement

The authors declare that the research was conducted in the absence of any commercial or financial relationships that could be construed as a potential conflict of interest.

## Footnotes

^1^https://senselab.med.yale.edu/modeldb/ShowModel.asp?model=149914
